# Query

**Published:** 1876-03

**Authors:** 


### QUERY.

BY NEMO.

In the January number of the REGISTER, the following question is asked:—"Have the eyes and teeth any nervous connection?"

Reflex nervous action is the key to the whole physiology of the nervous system. A knowledge of which will make plain many abnormal conditions in pathology, which cannot be explained in any other way, and which abnormal condition can be relieved or cured by proper treatment of the cause.

All dentists who have industry and mind enough to carry their investigations beyond effects to causes, will be forced to admit that uterine irritation, hepatic derangements, dentition, etc., etc., in certain instances, will induce pain and paralysis in remote parts of the body without apparent lesion. A morbid impression from injnry or disease in one part of the body is transmitted along a nerve to the nerve center. And if we were to be governed in all cases in the treatment of odontalgia by attacking the locality of the pain, we would often make unpardonable mistakes.

I will cite attention to two cases to show how reflex action, sympathy, transfer of nervous irritation, or whatever you may be pleased to call it, can produce disturbance in a remote part of the body. The case of H. Jones' where strabismus from palsy of the external rectus oculi muscle, disappeared after a piece of dead bone had been extracted from a whitlow on the thumb. And Lawrence's, in which blindness of one eye for thirteen months duration, was cured by the extraction of a carious tooth, with a splinter of wood projecting from one of the fangs.

Cases in practice. Miss C., aged 25 years, healthy, and teeth better than ordinary, was afflicted at times for months with paralysis of the left arm. During the time, she was under the treatment of an able physician, without any benefit. In conversation with him, how diseased teeth could affect remote parts of the body, he expressed a wish to have his patient's teeth examined. The nerve of the left superior wisdom tooth was found exposed; it was at once extracted and the palsy was cured. She had experienced no unusual sensation about her teeth.

Case 2d. Mrs. L., aged 23, and married three years, shortly after marriage, epilepsy developed itself; previously she had enjoyed good health. After treatment by different physicians, without getting better, but rather worse, and having become much reduced, she consulted one of reputed originality, who after a careful diagnosis, informed her the first thing to be done was the extraction of all the diseased teeth she had; which included all, except the eight inferior anterior ones, after which he put her on a course of tonics and regimen. A cure was the result.

Case 3d. Mr. B., has had odontalgia in the left first superior molar tooth, cured three times within the last three years, by the use of Comp. Cathartic Pills.

Case 4th. Mrs. G., aged about 25 years, never had any children, suffered much from tooth ache. Her physician and dentist, as her teeth and their appendages presented a normal appearance, were unable to assign a cause for her suffering. After canvassing her case, the question was asked if her tooth ache was not periodic, and occurred at the time of her catamanea. Her answer was—Yes.

